# Talc slurry versus thoracoscopic talc insufflation for malignant pleural effusion: a systematic review and meta-analysis

**DOI:** 10.36416/1806-3756/e20240115

**Published:** 2024-07-29

**Authors:** Anna Luíza Soares de Oliveira Rodrigues, Maria Eduarda Cavalcanti Souza, Francisco Cezar Aquino de Moraes, David Paes de Lima, Rafael Lucas Costa de Carvalho

**Affiliations:** 1. Centro Universitário de João Pessoa, João Pessoa (PB) Brasil.; 2. Universidade de Pernambuco, Recife (PE) Brasil.; 3. Universidade Federal do Pará, Belém (PA) Brasil.; 4. Divisão de Cirurgia Torácica, Instituto do Coração - InCor - Universidade de São Paulo (SP) São Paulo.

**Keywords:** Talc, Pleurodesis, Pleural effusion, malignant

## Abstract

**Objective:**

Talc pleurodesis is a widely used treatment option for malignant pleural effusion (MPE). However, the optimal form of administration remains controversial. Thus, we performed a systematic review and meta-analysis to assess the effectiveness of talc slurry (TS) in comparison with thoracoscopic talc insufflation/poudrage (TTI) for MPE treatment.

**Methods:**

We searched PubMed, EMBASE, and Cochrane Library databases for studies that compared TS with TTI in patients with MPE. We used a random-effects model with a 95% CI to pool the data. Heterogeneity was assessed with I^2^ statistics.

**Results:**

We included eight studies involving 1,163 patients, 584 of whom (50.21%) underwent TS. Pleurodesis failure rates were similar between the procedures (OR = 1.07; 95% CI: 0.56-2.06; p = 0.83; I^2^ = 62%); and 68% of patients (95% CI: 0.31-1.47; p = 0.33; I^2^ = 58%) had postoperative complications, which were lower in patients in the TS group than in the TTI group. In a subgroup analysis considering only randomized clinical trials, the failure rate was significantly lower in the TS treatment group (OR = 0.62; 95% CI: 0.42-0.90; p = 0.01; I^2^ = 0%). Similarly, dyspnea was less common in the TS group (OR = 0.74; 95% CI: 0.41-1.34; p = 0.32; I^2^ = 55%). Adverse effects were reported in 86 patients, and no significant difference was seen between the TS and TTI groups: empyema (OR = 1.43; 95% CI: 0.36-5.64; p = 0.86; I^2^ = 0%), pain (OR = 1.22 (95% CI: 0.67-2.21; p = 0.51; I^2^ = 38%), and pneumonia (OR = 1.15; 95% CI: 0.30-4.46; p = 0.86; I^2^ = 27%).

**Conclusions:**

Our findings suggest that TS is an effective treatment for MPE, with no significant increase in adverse events. Results suggest equivalent efficacy and safety for both procedures.

## INTRODUCTION

Malignant pleural effusion (MPE) is characterized by the presence of fluid and malignant cells in the pleural cavity.^1^ ^,^ ^2^ MPEs affect approximately up to 15% of all patients with cancer. Meanwhile, lung cancer and breast cancer account for 50-65% of MPEs,^2^ and more than 90% of patients with mesothelioma present with MPE.^3^ ^,^ ^4^ The incidence of MPE is likely to rise as the global incidence of cancer increases and overall survival improves.^5^ Regardless of the moment of presentation, the presence of MPE usually portends a poor prognosis.^5^ ^,^ ^6^ The clinical manifestation spectrum varies according to the severity of the effusion as well as with individual characteristics.^7^ ^,^ ^8^ The majority of patients with MPE are symptomatic, with debilitating symptoms, such as breathlessness, which is the most common symptom, or chest pain.^4^ In the presence of MPE, an intervention is required along with cancer treatment.^9^

Treatment options are determined by the patient’s clinical status, the type of tumor itself, the response to systemic therapy, and the degree of lung re-expansion following pleural fluid evacuation. The more traditional and established approach to MPE is pleurodesis.^10^ ^-^ ^13^ Pleurodesis is a procedure that obliterates the pleural space to prevent recurrent pleural effusion. Once the pleural cavity is evacuated, further fluid formation is commonly prevented by stimulating a local inflammatory response, resulting in fibrosis and adhesion, by either instilling a chemical irritant (chemical pleurodesis) or performing mechanical abrasion. According to international guidelines, talc is the preferred agent used for chemical pleurodesis.^6^ The primary perceived benefit of this approach is that a single intervention can lead to long-term fluid prevention, and the estimated success rate ranges from 80% to 100%.^12^ ^,^ ^13^

Talc slurry (TS) via chest tube is the current standard treatment approach for pleurodesis. Usually, TS requires the insertion of a chest tube to administer the chemical substance. An alternative method, known as thoracoscopic talc poudrage or insufflation (TTI), is the application of sterile talc powder under direct visualization during thoracoscopy.^13^ Evidence of high quality for the optimal treatment of symptomatic MPE suggests that both talc pleurodesis procedures (via slurry or poudrage) are highly effective and significantly improve symptoms.^4^ Meanwhile, other studies reported fewer recurrence rates with TTI.^13^ However, there is still uncertainty regarding whether TTI is more beneficial when compared with TS. Therefore, we conducted a systematic review and meta-analysis aiming to compare TTI with TS regarding pleurodesis in patients with MPE.

## METHODS

### Protocol and registration

This systematic review and meta-analysis followed the Preferred Reporting Items for Systematic Reviews and Meta-Analysis (PRISMA) guidelines. The protocol was prospectively registered in the International Prospective Register of Systematic Reviews (PROSPERO) under the registration number CRD42023414497.

### Eligibility criteria

Studies that met the following eligibility criteria were included: (1) randomized controlled trials (RCTs) or observational studies; (2) comparing treatment via TS with TTI treatment; (3) in individuals with MPE. We excluded studies (1) with overlapping populations; (2) not reporting outcomes of interest; or (3) unpublished results.

### Search strategy and data extraction

PubMed, Cochrane Library, and EMBASE were systematically searched on May 22, 2023. The search strategy included the terms chemical pleurodesis, pleurodesis, talc pleurodesis, surgical pleurodesis, thoracoscopic pleurodesis, thoracoscopic talc pleurodesis, thoracoscopic poudrage, thoracoscopic talc poudrage, talc insufflation, thoracoscopic talc insufflation, medical thoracoscopy, talc poudrage, bedside pleurodesis, medical pleurodesis, talc slurry, tube thoracostomy, chest tube talc slurry, chest tube, malignant pleural effusion, oncological patients. In addition, reference lists of included articles and previous systematic reviews were evaluated for additional eligible studies, and an alert was set for notifications in each database in case a new study correlated to the consultation carried out was eventually published.

All articles obtained from the initial literature search were entered into the reference management software Zotero, version 6 (Digital Scholar, Vienna, VA, USA). Duplicate articles were removed using both automated and manual methods. Subsequently, two authors (ALSOR and MECS) independently analyzed the titles and abstracts for inclusion criteria. Disagreements were resolved by consensus between the two authors and the senior author.

The following baseline characteristics were extracted: (1) ClinicalTrials.gov Identifier and study design; (2) number of patients allocated to each arm; (3) regimen details in experimental and control arms; and (4) main characteristics of patients. The same two authors collected the pre-specified baseline characteristics and outcome data.

### Endpoints and subgroup analysis

Outcomes of interest were as follows: (1) pleurodesis failure; (2) postoperative complications; (3) dyspnea; (4) respiratory complications; (5) empyema; (6) pain; (7) pneumonia; (8) postoperative death; (9) pulmonary edema; (10) reexpansion pulmonary edema; (11) fever; and (12) wound infection.

To minimize potential confounding factors due to selection bias or different prognostic factors, a post-hoc subgroup analysis including only RCTs was conducted. Studies reporting the failure rate of the procedure were included, and the criteria for its evaluation varied among the studies. Specifications of the criteria considered by each author are described in Table 1.

**Table 1:** Design and characteristics of studies included in this meta-analysis.

Study	Design	Follow-up (median)	Participants IG/CG, n.	Male+female/male, n.	Cancer type, n (%)		Successful pleurodesis in TS n/N, (%)	Successful pleurodesis in TTI n/N, (%)
					IG (TS)	CG (TTI)		
Bhatnagar et al.^2^	RCT	1-6 months	164/166	181/149	Lung - 54 (33) Breast - 49 (30) Other* - 57 (34.76) Unknown - 4 (2)	Lung - 59 (36) Breast - 50 (30) Other* - 54 (32.53) Unknown - 3 (2)	121/159 (76.10)	125/161 (77.64)
Dresler et al.^21^	RCT	1-6 months	240/242	269/213	Lung - 13 (26.5) Breast - 9 (18.5) Mesothelioma - 8 (16.5) Other* - 8 (16.5) Unknown - 11 (22)	Lung - 1 (4.5) Mesothelioma - 16 (73) Other* - 4 (18.19) Unknown - 2 (9)	80 (77.67)	38/49 (78.00)
Terra et al.^24^	RCT	1-6 months followed by every 3 months or if symptoms arose	30/30	45/15	Lung - 6 (20) Breast - 19 (63.34) Other* - 4 (13.34) Unknown - 1 (3.34)	Lung - 11 (36.67) Breast - 15 (50) Other* - 3 (10) Unknown - 1 (3.34)	26/30 (86.6)	25/30 (83.3)
Yim et al.^25^	RCT	6 weeks from 1-4.5 months, then every 3 months	29/28	37/20	Lung - 15 (53.57) Breast - 9 (32.14) Gastrointestinal - 4 (14.29) Other* - 1 (3.57)	Lung - 18 (62.07) Breast - 6 (20.69) Gastrointestinal - 2 (6.90) Other* - 2 (6.90)	27/28 (96.48)	26/29 (89.65)
Alihodzic-Pasalic et al.^8^	Retrospective	2.6 months	30/30	16/44	N/A	N/A	13/24 (54.16)	14/16 (87.50)
Debeljak et al.^20^	Retrospective	1 month	49/22	N/A	Lung - 13 (26.5) Breast - 9 (18.5) Mesothelioma - 8 (16.5) Other* - 8 (16.5) Unknown - 11 (22)	Lung - 1 (4.5) Mesothelioma - 16 (73) Other* - 4 (18.19) Unknown - 2 (9)	38/41 (92.68)	17/21 (80.95)
Inoue et al.^22^	Prospective	1-6 months	49/8	39/18	Lung - 28 (49.12) Breast - 15 (26.32) Ovarian - 4 (7.02) Others* - 10 (17.54)		40/45 (88.89%)	8/8 (100.0)
Stefani et al.^23^	Prospective	1, 3, and 6 months; monthly for 3 months	37/72	58/51	Lung - 21 (56.76) Breast - 7 (18.92) Other* - 9 (24.32)	Lung - 28 (38.89) Breast - 21 (29.17) Other* - 23 (31.95)	23/37 (62.16)	59/72 (81.95)

IG: intervention group; CG: control group; TS: talc slurry; TTI: thoracoscopic talc insufflation; RCT: randomized controlled trial. *Other: mesothelioma, lower gastrointestinal tract, kidney, upper gastrointestinal tract, lymphoma, osteosarcoma (tibia), pancreatic adenocarcinoma, adenocarcinoma of unknown origin, genitourinary, gynecologic, sarcoma, head and neck, and melanoma.

### Risk of bias assessment

The quality assessment of each RCTs was carried out using the Cochrane Collaboration tool for assessing the risk of bias in randomized trials (RoB 2) and nonrandomized studies were assessed using the Risk of Bias in Nonrandomized studies of intervention (ROBINS I).^14^ ^,^ ^15^ For each randomized trial, a risk of bias score was assigned, indicating whether it was at a high, low, or unclear risk across five domains: randomization process, deviations from intended interventions, missing outcomes, measurement of outcomes, and selection of reported results. To assess publication bias, funnel-plot analyses were employed.^16^ In this assessment, each study was categorized as critical, serious, moderate, or low risk in the seven domains: confounding, selection, classification, deviations from intended interventions, missing data, and selection of reported results. Two authors (FCAM and MECS) independently conducted the assessment, and disagreements were resolved by consensus. To quantify publication bias, Begg and Mazumdar rank correlation and Egger’s linear regression methods were used.

### Statistical analysis

Binary endpoints were evaluated with hazard ratios (HRs) or odds ratios (ORs), with 95% CIs. The Cochran’s Q-test and I^2^ statistics were used to assess heterogeneity; p values > 0.10 and I^2^ values > 25% were considered to indicate significance for heterogeneity.^16^ We used DerSimonian and Laird random-effect models for all endpoints.^17^ ^,^ ^18^ Statistical analyses were performed using R statistical software, version 4.2.3 (R Foundation for Statistical Computing, Vienna, Austria).

## RESULTS

### Study selection and baseline characteristics

**Figure 1:**
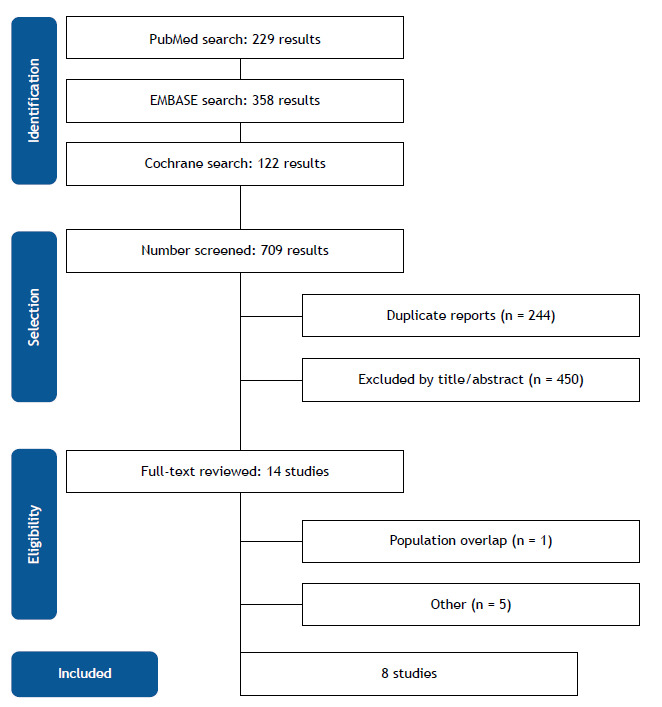
Preferred Reporting Items for Systematic Review and Meta-Analysis (PRISMA) flow diagram of study screening and selection.

As illustrated in Figure 1, the initial search strategy yielded 709 articles, of which 244 were excluded after title and abstract review and removal of duplicate reports. The remaining were fully reviewed, and eight studies were included in this meta-analysis.^19^ ^-^ ^25^ The studies included involved 1,163 patients (Figure 1). Among them, 584 (50.21%) underwent TS treatment. Mean follow-up period ranged from 1 to 6 months. Among studies that reported the cancer type, the most prevalent types were lung (n = 410; 33.5%) and breast (n = 379; 31%) cancer, while other types accounted for 33.5%. Study and participant characteristics are summarized in Table 1. The definition of success varied across studies, and specific details regarding success criteria for each study can be found in Table S1 of the supplementary material, and the definition of failure can be found in Table S2.

### Pooled analyses of all studies

**Figure 2:**
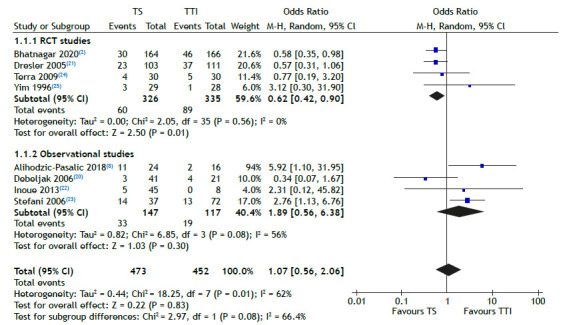
Treatment failure rate (pleurodesis) using talc slurry versus thoracoscopic talc insufflation, also known as thoracoscopic talc poudrage, in patients with malignant pleural effusion.

Regarding pleurodesis failure rate, this analysis showed no significant difference between TS and TTI groups (OR = 1.07; 95% CI: 0.56-2.06; p = 0.83; I^2^ = 62%; Figure 2). Likewise, there were no significant differences between the groups in terms of postoperative complications, post-operative death, and pulmonary edema (Table 2). Regarding other adverse events, there were no significant statistical differences between TS and TTI treatment in regard to pneumonia, empyema, dyspnea, pain, fever, reexpansion pulmonary edema, and wound infection (Table 2).

**Table 2:** Statistical analysis of the adverse events of interest.

Adverse events	Study, n	Patients, n	OR	95% CI	p	Heterogeneity			
						T^2^	df	p	I^2^ (%)
Dyspnea	3	809	0.74	0.41-1.34	0.32	0.15	2	0.11	55
Empyema	4	659	1.43	0.36-5.64	0.97	0.00	3	0.86	0
Fever	4	445	1.13	0.73-1.75	0.59	0.00	3	0.45	0
Pain	4	929	1.22	0.67-2.21	0.18	0.14	3	0.18	38
Pneumonia	3	499	1.15	0.30-4.46	0.84	0.48	2	0.26	27
Postoperative complications	3	461	0.68	0.31-1.47	0.33	0.28	2	0.09	58
Postoperative death	7	1,103	0.87	0.60-1.27	0.48	0.00	2	0.62	0
Pulmonary edema	4	297	0.35	0.08-1.63	0.18	0.00	3	0.84	0
Reexpansion pulmonary edema	3	226	1.51	0.42-5.39	0.52	0.00	2	0.86	0
Wound infection	4	440	1.29	0.26-6.48	0.76	0.00	2	0.93	0

df: degrees of freedom.

The rate of side effects was comparable in the TS and TTI treatment groups within the trials. Overall, fever was the most prevalent adverse effect, with 193 events (47.67% vs. 52.33%). When analyzing organism system disorders, 2 patients had a cerebrovascular event (100% vs. 0%) as the most frequent nervous disorder, 25 had dysrhythmia or arrhythmia (44% vs. 56%) as a cardiovascular disorder, 77 had pneumonia (38.96% vs. 61.04%) as a respiratory disorder, 4 had nausea or vomiting (50% vs. 50%) as a gastrointestinal disorder, and 18 had emphysema (27.78% vs. 72.22%) as the most prevalent tissue disorder. There were a total of 43 events leading to death (39.53% vs. 60.46%) and 158 post-operative deaths (46.84% vs. 53.16%). The results for adverse events are detailed in Table 2 and in the supplementary material (Figures S1-S10).

### Subgroup analysis

In the analysis of only RCTs, which involved 661 patients, the failure rate was significantly lower in the TS treatment group (OR = 0.62; 95% CI: 0.42-0.90; p = 0.01; I^2^ = 0%; Figure 2). Additionally, dyspnea was less common in the TS group (OR = 0.74; 95% CI: 0.41-1.34; p = 0.32; I^2^ = 55%; Table 2). Furthermore, 86 patients reported other adverse events, but there were no significant differences between the TS and TTI groups in terms of empyema, pain, and pneumonia (Table 2).

### Quality assessment

**Figure 3:**
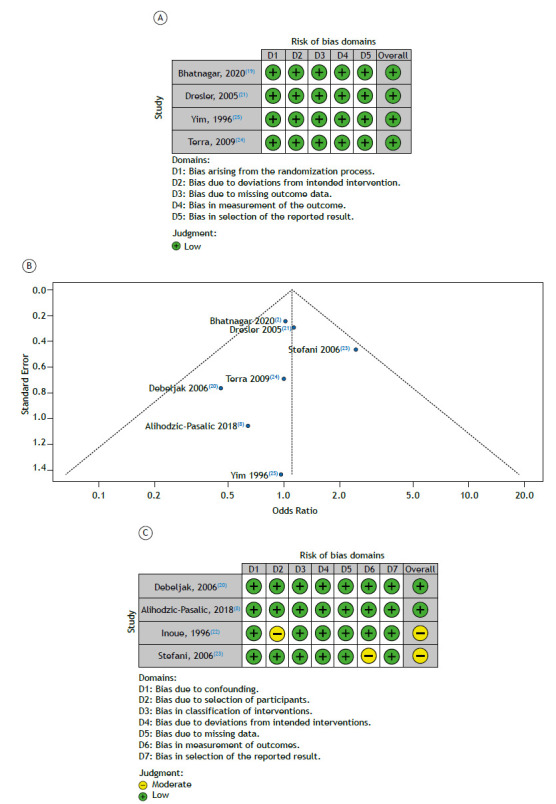
In A, critical appraisal of randomized controlled trials according to the Cochrane Collaboration’s tool for assessing risk of bias in randomized trials (RoB 2). In B, results of the Risk Of Bias in Nonrandomised Studies of Interventions (ROBINS-I) regarding the observational studies included in the analysis. In C, funnel plot analysis of treatment failure rate (pleurodesis) using talc slurry versus thoracoscopic talc insufflation in patients with malignant pleural effusion. There is no evidence of publication bias.

Our meta-analysis included 4 RCTs and 4 observational studies. The assessment of the RCTs demonstrated a low risk across all studies (Figures 3A and 3B). Among the included nonrandomized studies, two presented one domain with a moderate risk of bias, while other domains were labeled as with a low risk. The funnel plot analysis showed an asymmetry in the distribution of studies according to the failure rate (Figure 3C), although no significant publication bias was detected by Egger’s (p = 0.1471) and Begg and Mazumdar tests (p = 0.3272).

## DISCUSSION

In this systematic review and meta-analysis of eight studies including 1,163 patients, we compared TS pleurodesis with TTI in patients with MPE. The main finding from the analysis is that there was no significant difference between treatments regarding failure rates when analyzing randomized and nonrandomized studies. Similarly, Bhatnagar et al.^19^ and Dresler et al.^21^ concluded that both approaches are effective. However, when pooling subgroup data only from RCTs to minimize selection bias, the results indicated that TS was associated with a lower failure rate.

In clinical practice, the treatment of choice is based on several factors, including whether a chest tube has already been inserted; the infrastructure of the local facility where the treatment will take place; staff experience and training; and patient phenotype regarding fluid production and accessibility.^4^ ^,^ ^5^ ^,^ ^8^ ^,^ ^26^ Only one observational study explained the characteristics of the patients that were used in order to select the groups.^8^ Alihodzic-Pasalic et al. selected those patients at high risk for general anesthesia into the TS group.^(8^

The benefits of performing thoracoscopy are that it allows the surgeon to examine the pleural cavity and perform a pleural biopsy or adhesiolysis,^9^ and it is most often the preferred choice of physicians.^27^ ^-^ ^30^ Additionally, TS offers additional advantages, such as the possibility of administration in patients who are not candidates for surgery, but still allows diagnostic procedures such as pleural biopsy needle biopsy. This flexibility is crucial, especially in cases in which surgery is not a viable option, but therapeutic or diagnostic interventions in the pleural cavity are necessary.^6^ ^,^ ^7^ ^)^ However, previous observational studies addressing the ideal method for administering talc diverged in their results and were considered inconclusive, resulting in inconsistency in both clinical practice and recommendations.^19^ ^,^ ^27^

This meta-analysis showed lower failure rates in the TS group, when compared with the TTI group, when only RCT data were pooled, which supports that talc pleurodesis performed at the bedside through a chest tube is more effective than TTI. Our results differ from previous meta-analyses. Mummadi et al. suggest that there is no difference between the techniques.^27^ Beltsios et al. compared talc pleurodesis with other approaches, and a statistically significant superiority was seen when compared with control methods, especially when compared with bleomycin.^29^

Adverse effects may occur due to inflammation of the pleura by the agent chosen for pleurodesis. In our analysis, there was no statistical difference in the occurrence of adverse effects, such as dyspnea, respiratory complications, empyema, pain, pneumonia, postoperative death, pulmonary edema, reexpansion pulmonary edema, fever, and wound infection. Likewise, the occurrence of postoperative complications was statistically similar in both treatment groups.

This study has some limitations. Firstly, we included both RCT and observational study data, which can introduce bias. However, the implementation of a subgroup analysis of only RCTs was possible. This approach was aimed at mitigating potential confounding factors. Secondly, different criteria were used for evaluating success and failure rates among the studies. Thirdly, the type of cancer varied among the study populations, and, in some studies, certain details of population characteristics, such as the size of the effusion, were not provided. Fourth, not all observational studies characterized the conditions of patients and whether these were choice points for selection between intervention and control groups. Although the heterogeneity was high for the main outcome in the analysis of all included studies, that was not significant, and when pooling data only from RCTs, the heterogeneity was low.

## FINAL CONSIDERATIONS

In this meta-analysis, the use of TS for MPE treatment demonstrated comparable failure rates to the use of TTI without a significant increase in adverse events. These results suggest that both interventions are equally effective and safe for managing MPE, aligning with the overall findings of the primary analysis.

## References

[1] Desai NR, Lee HJ (2017). Diagnosis and management of malignant pleural effusions state of the art in 2017. J Thorac Dis.

[2] Bhatnagar R, Laskawiec-Szkonter M, Piotrowska HE, Kahan BC, Hooper CE, Davies HE (2014). Evaluating the efficacy of thoracoscopy and talc poudrage versus pleurodesis using talc slurry (TAPPS trial) protocol of an open-label randomised controlled trial. BMJ Open.

[3] Psallidas I, Kalomenidis I, Porcel JM, Robinson BW, Stathopoulos GT (2016). Malignant pleural effusion from bench to bedside. Eur Respir Rev.

[4] Bibby AC, Dorn P, Psallidas I, Porcel JM, Janssen J, Froudarakis M (2018). ERS/EACTS statement on the management of malignant pleural effusions. Eur Respir J.

[5] Feller-Kopman DJ, Reddy CB, DeCamp MM, Diekemper RL, Gould MK, Henry T (2018). Management of Malignant Pleural Effusions An Official ATS/STS/STR Clinical Practice Guideline. Am J Respir Crit Care Med.

[6] Roberts ME, Neville E, Berrisford RG, Antunes G, Ali NJ, BTS Pleural Disease Guideline Group (2010). Management of a malignant pleural effusion British Thoracic Society Pleural Disease Guideline 2010. Thorax.

[7] Skok K, Hladnik G, Grm A, Crnjac A (2019). Malignant Pleural Effusion and Its Current Management A Review. Medicina (Kaunas).

[8] Alihodzic-Pasalic A, Maric V, Hadzismailovic A, Pilav A, Grbic K (2018). Comparison of Efficiency of Pleurodesis Between Video Assisted Thoracoscopic Surgery (VATS) and Standard Thoracostomy. Acta Inform Med.

[9] Rahman NM, Ali NJ, Brown G, Chapman SJ, Davies RJ, Downer NJ (2010). Local anaesthetic thoracoscopy British Thoracic Society Pleural Disease Guideline 2010. Thorax.

[10] Basso SM, Mazza F, Marzano B, Santeufemia DA, Chiara GB, Lumachi F (2012). Improved quality of life in patients with malignant pleural effusion following videoassisted thoracoscopic talc pleurodesis Preliminary results. Anticancer Res.

[11] Davies HE, Lee YC (2013). Management of malignant pleural effusions questions that need answers. Curr Opin Pulm Med.

[12] Lumachi F, Mazza F, Ermani M, Chiara GB, Basso SM (2012). Talc pleurodesis as surgical palliation of patients with malignant pleural effusion Analysis of factors affecting survival. Anticancer Res.

[13] Heffner JE, Klein JS (2009). Recent advances in the diagnosis and management of malignant pleural effusions [published correction appears in Mayo Clin. Proc.

[14] Sterne JAC, Savovic J, Page MJ, Elbers RG, Blencowe NS, Boutron I (2019). RoB 2 a revised tool for assessing risk of bias in randomised trials. BMJ.

[15] Sterne JA, Hernán MA, Reeves BC, Savovic J, Berkman ND, Viswanathan M (2016). ROBINS-I a tool for assessing risk of bias in non-randomised studies of interventions. BMJ.

[16] Egger M, Davey Smith G, Schneider M, Minder C (1997). Bias in meta-analysis detected by a simple, graphical test. BMJ.

[17] DerSimonian R, Laird N (1986). Meta-analysis in clinical trials. Control Clin Trials.

[18] IntHout J, Ioannidis JP, Borm GF (2014). The Hartung-Knapp-Sidik-Jonkman method for random effects meta-analysis is straightforward and considerably outperforms the standard DerSimonian-Laird method. BMC Med Res Methodol.

[19] Bhatnagar R, Piotrowska HEG, Laskawiec-Szkonter M, Kahan BC, Luengo-Fernandez R, Pepperell JCT (2020). Effect of Thoracoscopic Talc Poudrage vs Talc Slurry via Chest Tube on Pleurodesis Failure Rate Among Patients With Malignant Pleural Effusions A Randomized Clinical Trial. JAMA.

[20] Debeljak A, Kecelj P, Triller N (2006). Talc pleurodesis comparison of talc slurry instillation with thoracoscopic talc insufflation for malignant pleural effusions. J BUON.

[21] Dresler CM, Olak J, Herndon 2nd JE, Richards WG, Scalzetti E, Fleishman SB (2005). Phase III intergroup study of talc poudrage vs talc slurry sclerosis for malignant pleural effusion. Chest.

[22] Inoue T, Ishida A, Nakamura M, Nishine H, Mineshita M, Miyazawa T (2013). Talc pleurodesis for the management of malignant pleural effusions in Japan. Intern Med.

[23] Stefani A, Natali P, Casali C, Morandi U (2006). Talc poudrage versus talc slurry in the treatment of malignant pleural effusion A prospective comparative study. Eur J Cardiothorac Surg.

[24] Terra RM, Junqueira JJM, Teixeira LR, Vargas FS, Pêgo-Fernandes PM, Jatene FB (2009). Is full postpleurodesis lung expansion a determinant of a successful outcome after talc pleurodesis?. Chest.

[25] Yim AP, Chan AT, Lee TW, Wan IY, Ho JK (1996). Thoracoscopic talc insufflation versus talc slurry for symptomatic malignant pleural effusion. Ann Thorac Surg.

[26] Davies HE, Mishra EK, Kahan BC, Wrightson JM, Stanton AE, Guhan A (2012). Effect of an indwelling pleural catheter vs chest tube and talc pleurodesis for relieving dyspnea in patients with malignant pleural effusion the TIME2 randomized controlled trial. JAMA.

[27] Mummadi S, Kumbam A, Hahn PY (2014). Malignant pleural effusions and the role of talc poudrage and talc slurry a systematic review and meta-analysis. F1000Res.

[28] Light RW (2012). Counterpoint should thoracoscopic talc pleurodesis be the first choice management for malignant pleural effusion? No. Chest.

[29] Beltsios ET, Mavrovounis G, Adamou A, Panagiotopoulos N (2021). Talc pleurodesis in malignant pleural effusion a systematic review and meta-analysis. Gen Thorac Cardiovasc Surg.

[30] Lee P (2012). Point Should thoracoscopic talc pleurodesis be the first choice management for malignant effusion? Yes. Chest.

